# Children's rights and restrictive measures during the COVID-19 pandemic: implications for politicians, mental health experts and society

**DOI:** 10.1186/s13034-023-00617-8

**Published:** 2023-06-21

**Authors:** Jörg M. Fegert, Helena Ludwig-Walz, Andreas Witt, Martin Bujard

**Affiliations:** 1 Department for Child and Adolescent Psychiatry and Psychotherapy, University Medical Center Ulm, Ulm, Germany; 2 Competence Area Mental Health Prevention in the Competence Network Preventive Medicine Baden-Württemberg, Ulm, Germany; 3 Federal Institute for Population Research (BiB), Wiesbaden, Germany; 4 University Psychiatric Services Bern (UPD), Bern, Switzerland; 5 Institute of Medical Psychology, Medical Faculty, University Heidelberg, Heidelberg, Germany

The COVID-19 pandemic extends widely beyond the purely physical aspects (infection, recovery, medical treatment), ultimately encompassing nearly all fields of health in all age classes and social interactions. Concurrently, the pandemic management revealed that policy decisions in many countries around the world did not adequately consider the impact of these measures on the development of children and adolescents. Maintaining child care and schooling are necessary for many children and adolescents, not only in terms of their educational opportunities, but also in terms of promoting their mental health. Two meta-analyses of depression (Ludwig-Walz et al. 2022) and anxiety (Ludwig-Walz et al. 2023) as a result of the COVID-19 pandemic show the clear links between restrictive policies, particularly school closures, and the rise of depression and anxiety symptoms in children and adolescents.

While various lobby groups were able to ensure that production and commerce could continue as well as possible, it was again shown that children and adolescents do not have a sufficient lobbying presence. A great deal of restrictions was imposed on younger people in order to protect the particularly vulnerable older generation. Developmental tasks typical of their age, which include going out and making friends for the first time, as well as moving on to vocational training or university studies, were much more difficult to accomplish, and only with considerable restrictions.

In this thematic series "child and adolescent mental health during the Covid-19 pandemic" in CAPMH, psychological stress during COVID-19 and issues of sickness treatment during pandemic conditions have been repeatedly addressed. The current bottlenecks and increased need for curative treatment of children and adolescents with mental stress were foreseeable (cf. Fegert et al. 2020). In 2023, if we want to talk about lessons learned, it is also important to note the strong psychosocial impact that ‘normal’ daily life and school attendance have on children and adolescents.

Therefore, it is all the more incomprehensible why for a long time, the various crisis management units at different levels did not consider child mental health to be a central issue and why experts from this field were not consulted when it came to designing restrictions. Initiating interdisciplinary expert task forces would be indispensable, not only for controlling and managing the COVID-19 pandemic, but also for returning to youth’s normal everyday life. In addition to politics and lobbying, the role of mass media has to be mentioned: Until summer 2021, after the second-long lockdown phase, the majority of mass media ignored mental health consequences of school closures while emphasizing nearly every day COVID-19 incidences and the infection risks of children and adolescents. However, analyses for the first lockdown not only found a significant increase in depression compared to the pre-pandemic baseline, but also a high magnitude from an epidemiological perspective. Extrapolation analyses based on panel data and demographic statistics for Germany, where school closures and pandemic-related restrictions were more stringent, showed that the increase of clinically relevant depression symptoms during the first pandemic lockdown applied to approximately 477,000 adolescents aged 16–19 and a reduced health-related quality of life (HRQoL) for 1.7 million children aged 11–17 (Bujard et al. 2021). For politicians and the media, those absolute numbers are often more convincing than the significance of results, especially since absolute numbers were often used in the discourse on COVID-19 incidences and deaths.

The "long way back to normality" that was already suspected at the beginning of the pandemic, based on experience from local epidemics and the last global economic crisis, is now becoming regrettably apparent. A Europe-wide survey conducted by the McKinsey Health Institute (Fegert and Deetjen 2023) revealed high levels of self-perceived mental distress, considerable difficulties in accessing help and a high tendency of self-stigmatization in dealing with one’s own perceived mental problems for Generation Z, i.e. young people who are now in the transition to adulthood, despite considerable differences.

At the same time, a particular reluctance was noted to visit specialized psychological centres and established child and adolescent psychotherapists or psychiatrists. Participants from GenZ Mc Kinsey Health survey formulated their greatest expectations for peer support. However, peers can quickly become overwhelmed by problems such as suicidality. Nevertheless, in many countries, waiting times for consultation have increased, which has led to increased triage of patients. The accumulation of consecutive crises changes the perception of personal future prospects, especially in Europe with the ongoing war in Ukraine but also with climate change, which many young people worldwide clearly perceive as a threat to their own life chances (Arora et al. 2022). Also, the overlap of the aforementioned various mental stressors can inevitably increase the severity and extent of mental health disorders.

Thus, from a child and adolescent psychiatric perspective, child and adolescent mental health is a societal challenge and responsibility that far exceeds the mere issue of patient care and treatment. Some of the now reported findings of an increase in depression and anxiety symptoms, in connection with COVID-19 restrictions (Ludwig-Walz et al. 2022, Ludwig-Walz et al. 2023) indicate a general increase in the burden of internalizing symptoms, but not a real increase in disorders requiring treatment. Here, we must caution against interpreting average expectancies alone, since during the pandemic years we were all discouraged with respect to social contacts, large cultural and sporting events, etc., and thus tend to react cautiously and somewhat anxiously in new exposure situations. Wherever the symptoms have actually led to social withdrawal and to an impairment of participation, i.e. to symptoms requiring treatment, it is to be feared that the mental disorders that have arisen will also become chronic in the natural course with the same risk as before the pandemic. This would result in much higher levels of chronic mental health disorders than before the pandemic.

We have to emphasize, however, that mental disorders already accounted for a large proportion of the burden of disease in young people before the pandemic and only limited treatment was available or was often accompanied by stigmatization. In this respect, the health care system in many countries in Europe and the global north face a threat of a large wave of socially impairing burdens requiring treatment over the next decades.

It is therefore all the more important to take preventive action whenever possible. Distinguishing between three levels of prevention is well established in public health (e.g. Gordon 1983): Universal, selective and indicated prevention. With universal prevention information and advice is directed at the entire population, in particular at parents and those working with children and adolescents such as teachers, sport trainers etc. Selective prevention is targeted at special risk groups such as children and adolescents who were in transition phases during the pandemic, e.g. in the transition from kindergarten to school, from elementary school to secondary education or in the transition to adulthood. For the children and adolescents during the pandemic, it was particularly difficult to grow into new peer groups. It seems especially urgent to strengthen social cohesion so that normal developmental tasks can be mastered. Of special importance is indicated prevention, i.e. targeted, evidence-based early interventions for children and adolescents where school nurses/social workers, peers or other bystanders such as the parents of classmates have already identified a change or need for help. Indicated prevention is at the threshold of early intervention. Thus, in a stepped-care approach it can be avoided as early as possible that the increase of symptoms that can be observed at this stage actually becomes a manifest impairment in everyday life and a chronic mental health problem via the psychosocial consequences such as social withdrawal, etc.

Proactive programmes should be launched and evaluated not only in the health care sector. Evidence based or best practice models should be made available for a large audience. Therefore, CAPMH continues to provide a platform for research in the field and welcomes submissions in the ongoing series on "child and adolescent mental health during the Covid-19 pandemic". This requires interdisciplinary collaboration and the initiation of a general monitoring of children and adolescents who experienced the pandemic in a particularly intense way. Also needed are resolute medium- and long-term assessments of mental health disorders as a foundation for conceptualizing targeted programmes. In the sense of intergenerational justice, it is an ethical obligation [cf. ad hoc recommendation of the German Ethics Council (2022)] to give back to the younger generation—a generation which greatly contributed to the safety of older people and people at risk during the acute pandemic.

## Data Availability

Not applicable.
