# Correction: Children’s rights and restrictive measures during the COVID-19 pandemic: implications for politicians, mental health experts and society

**DOI:** 10.1186/s13034-023-00641-8

**Published:** 2023-07-17

**Authors:** Jörg M. Fegert, Helena Ludwig-Walz, Andreas Witt, Martin Bujard

**Affiliations:** 1 Department for Child and Adolescent Psychiatry and Psychotherapy, University Medical Center Ulm, Ulm, Germany; 2 Competence Area Mental Health Prevention in the Competence Network Preventive Medicine Baden-W?rttemberg, Ulm, Germany; 3 Federal Institute for Population Research (BiB), Wiesbaden, Germany; 4 University Psychiatric Services Bern (UPD), Bern, Switzerland; 5 Institute of Medical Psychology, Medical Faculty, University Heidelberg, Heidelberg, Germany

**Correction to: Child and Adolescent Psychiatry and Mental Health (2023) 17:75**

10.1186/s13034-023-00617-8

Following publication of the original article [1], the author noticed the errors occurred in the formatting of values and in the reference list. Now, these errors have been corrected with this erratum.

In fourth paragraph, there is a formatting error in the values which should be 477,000 instead of 4,77,000.

In first reference, the publication year has repeated twice which has been corrected now:

Arora et al. Heat waves, the war in Ukraine, and stigma: Gen Z’s perspectives on mental health. In: McKinsey and Company, 2022. https://www.mckinsey.com/mhi/our-insights/heat-waves-the-war-in-ukraine-andstigma-gen-zs-perspectives-on-mental-health (Accessed 18 April 2023).

The DOI link has been included for the below references:

Fegert JM, Vitiello B, Plener P, Clemens V. Challenges and burden of the Coronavirus 2019 (COVID-19) pandemic for child and adolescent mental health: a narrative review to highlight clinical and research needs in the acute phase and the long return to normality. Child Adolesc Psychiatry Ment Health. 2020;14:1–11. 10.1186/s13034-020-00329-3.

Ludwig-Walz H, Dannheim I, Pfadenhauer LM, Fegert JM, Bujard M. Increase of depression among children and adolescent after the onset of the COVID-19 pandemic in Europe: a systematic review and meta-analysis. Child Adolesc Psychiatry Ment Health. 2022;16:109. 10.1186/s13034-022-00546-y.

Ludwig-Walz H, Dannheim I, Pfadenhauer LM, Fegert JM, Bujard M. Anxiety increased among children and adolescents during pandemic-related school closures in Europe: a systematic review and meta-analysis. Child Adolesc Psychiatry Ment Health 2023;17:74. 10.1186/s13643-023-02225-1.

The original article has been corrected.
